# Extracorporeal Membrane Oxygenation Use, Expenditure, and Outcomes in Taiwan From 2000 to 2010

**DOI:** 10.2188/jea.JE20140027

**Published:** 2015-04-05

**Authors:** Chiao-Po Hsu, Wui-Chiang Lee, Hsiu-Mei Wei, Shih-Hsien Sung, Chun-Yang Huang, Chun-Che Shih, Tse-Min Lu

**Affiliations:** 1 Department of Medicine, School of Medicine, National Yang-Ming University, Taipei, Taiwan; 2 Division of Cardiovascular Surgery, Department of Surgery, Taipei Veterans General Hospital, Taiwan; 3 Institute of Hospital and Health Care Management, National Yang-Ming University School of Medicine, Taiwan; 4 Department of Medical Affairs and Planning, Taipei Veterans General Hospital, Taiwan; 5 Department of Healthcare Management, Yuanpei University, Taiwan; 6 Medical Record Management Section, Taipei Veterans General Hospital, Taiwan; 7 Division of Cardiology, Department of Internal Medicine, Taipei Veterans General Hospital, Taiwan; 8 Department of Cardiovascular Surgery, Far Eastern Memorial Hospital, Taipei, Taiwan

**Keywords:** cost, extracorporeal membrane oxygenation, survival, Taiwan

## Abstract

**Background:**

No study to date has systematically examined use, expenditure, and outcomes associated with extracorporeal membrane oxygenation (ECMO) use in Taiwan. The aim of this study was to examine ECMO use, expenditure, and outcomes during an 11-year period in Taiwan.

**Methods:**

Claims data were collected from the Taiwan National Health Insurance Research Database for patients who received ≥1 ECMO treatment between January 2000 and December 2010. Measurements included demographics, indications for ECMO use, length of hospital stay, outcome, and expenditure.

**Results:**

A total of 3969 patients received ECMO during the study period (median age: 54.6 years). The number of patients receiving ECMO increased from 52 in 2000 to 1045 in 2010. The major indication for ECMO was cardiovascular disease (68.7%), followed by respiratory disease (17.9%). Median length of hospital stay was 13 days in 2000 and 17 days in 2010. Median expenditure (New Taiwan dollars) was $604 317 in 2000 and $673 888 in 2010. Some variables significantly differed by age, sex, hospital setting, calendar year, and indication for ECMO, and were associated with in-hospital and after-discharge mortality.

**Conclusions:**

ECMO use has increased dramatically in Taiwan over the last decade. The high mortality rate of ECMO users suggested that ECMO may be being used in Taiwan for situations in which it provides no added benefit. This situation may be a reflection of the current reimbursement criteria for National Health Insurance in Taiwan. Refinement of the indications for use of ECMO is suggested.

## INTRODUCTION

Extracorporeal membrane oxygenation (ECMO) is a technique for providing cardiac and respiratory support to patients who have suffered cardiac or respiratory failure and have not responded to conventional therapy.^1^ Venovenous ECMO is used to provide respiratory support for patients with severe respiratory failure, whereas venoarterial ECMO can be used to provide respiratory and circulatory support.^2^ Both approaches are established interventions for the treatment of neonates with severe respiratory failure and as cardiorespiratory support for neonates after congenital heart defect repair.^3^^,^^4^ The use of ECMO in the treatment of adults with severe respiratory distress has been the topic of debate for some years.^3^^,^^4^ However, the findings from a multicenter randomized controlled trial (Conventional ventilation or ECMO for Severe Adult Respiratory failure [CESAR]) published in 2009 demonstrated that adults with severe respiratory failure who received ECMO had a significantly better survival outcome than those who received conventional support.^5^ Although the use of ECMO in adults is expected to increase because of the findings from CESAR,^2^ the benefits reported were clinically modest, and the cost-effectiveness of such treatment may not be applicable in all settings.^3^

In Taiwan, the cost of ECMO is reimbursed by the National Health Insurance (NHI) scheme, which covers 99.8% of the population. Specifically, since 2002, the NHI has reimbursed ECMO costs for the following indications: failure to wean from cardiopulmonary bypass after cardiac surgery, right heart failure due to reversible pulmonary hypertension crisis after cardiac surgery, end-stage heart disease waiting for heart transplantation, and reversible cardiomyopathy. In response to increasing numbers of requests from patients and their families, the indications were extended in 2009 to the treatment of adults with cardiogenic shock and respiratory failure due to certain illnesses, and to severe neonatal and pediatric illnesses (respiratory failure, meconium aspiration syndrome, hyaline membrane disease, and congenital diaphragm hernia). The increasing use (and costs) of ECMO and whether ECMO is being appropriately used in Taiwan are topics of intense debate.

Although the findings from a number of retrospective and observational studies suggest that the use of ECMO in Taiwan is effective and ethical,^6^^–^^13^ no study has systematically examined use, expenditure, and outcomes associated with ECMO in Taiwan. As most patients in Taiwan are covered by the NHI, this provides a unique opportunity to perform such a systematic study by extracting data from the NHI claims database. Therefore, the aim of this study was to examine ECMO use, expenditure, and patient outcomes in Taiwan between 2000 and 2010. We also performed analyses to identify factors associated with patient survival after ECMO.

## MATERIALS AND METHODS

### Study design and data sources

This was a retrospective, secondary analysis study of claims data from NHI beneficiaries who received ECMO at least once between 1 January 2000 and 31 December 2010.

For data analysis, we grouped the data into the time periods of 2000–2002, 2003–2008, and 2009–2010, because these time periods corresponded to periods which differed in NHI reimbursement criteria for ECMO. Indications for ECMO for each period are listed in Table 1. Data were obtained from the National Health Insurance Research Database (NHIRD), a large computerized database which has been available for public research use since 1999. The database contains original claims for reimbursement plus enrollment files of NHI beneficiaries. The enrollment files contain individual subscription information and demographic details, including sex, date of birth, type of beneficiaries, and geographic location of NHI enrollment. The claims files contain comprehensive and individual-level records of inpatient care, ambulatory care, pharmacy store, and dental care. The files also include the date of service, International Classification of Diseases, Ninth Revision, Clinical Modification (ICD-9-CM) diagnosis codes, claimed medical expenses, and the co-payment amount for each service. The identification number of patients and healthcare facilities in the datasets are encrypted to protect privacy before release for research purposes.

**Table 1.** Indications of ECMO in Taiwan

Before 2002/12/1: No National Health Insurance reimbursement		

ECMO indications 2002/12/1		
	1. Failure to wean from cardiopulmonary bypass after cardiac surgery	
	2. Right heart failure due to reversible pulmonary hypertension crisis after cardiac surgery	
	3. End-stage heart disease waiting for heart transplantation	
	4. Reversible cardiomyopathy	

Expanded indications of ECMO (2009/9/1)		
	1. Cardiogenic shock due to	
		a. Failure to wean from cardiopulmonary bypass after cardiac surgery, such as “stunned” heart
		b. As a bridge to either cardiac reconstruction operation, transplantation or placement of a ventricular assist device
		c. Reversible cardiomyopathy myocarditis
		d. Pulmonary embolism
		e. Severe acute myocardial infarction with cardiogenic shock
		f. Other types of cardiogenic shock
	2. Respiratory failure with	
		a. PaO_2_ <60 mm Hg in 100% O_2_, excluding any reversible factors
		b. CO_2_ retention and causing unstable hemodynamics, excluding any reversible factors
		c. As a bridge to lung transplantation
	3. Pediatric and neonatal	
	Primary diagnoses associated with a. Meconium aspiration syndrome, b. Hyaline membrane disease, c. Congenital diaphragm hernia, or d. Persistent pulmonary hypertension of neonate, and they meet the indices below (respiratory failure) despite the best accepted standard of care for management with a ventilator:	
		*Oxygenation Index ≥40*, Oxygenation Index = [(Mean airway pressure) × FiO_2_ × 100]/PaO_2_,
		*AaDO_2_ >610 mm Hg for 8 hours or >600 mm Hg for 12 hours*, AaDO_2_ = (Patm-47) × FiO_2_-PaO_2_-PaCO_2_/0.8,
	*PaO_2_ <40 mm Hg for 2 hours*	

	4. Other	
		a. Airway injury
		b. Profound hypothermia (core temperature ≤30°C)

For this study, we collected data from the NHIRD for NHI beneficiaries who had an ECMO ICD-9-CM diagnosis (39.65) or NHI service claim codes 68036A (before 2005) and/or 68036B (after June 2005). Claims data extracted included demographics, individual-level discharge diagnoses (ICD-9-CM codes) for each hospitalization, length of stay (LOS; in days), utilization codes for ECMO and other services, hospital setting (medical center, regional hospital, or district hospital), survival/mortality (in-hospital and after-discharge), NHI reimbursement, and copayment. In Taiwan, the vast majority of medical centers provide tertiary care and offer ECMO if clinically indicated. Regional hospitals can provide ECMO only if they have a strong cardiovascular team and intensive care capabilities. A limited number of district hospitals can provide ECMO. Regarding patient survival rates, some patients were discharged in critical condition so that they could die at home. These patients were coded as “advise against discharge” and, for the purposes of this study, were considered to have died after ECMO (in-hospital mortality), unless there were follow-up data to clearly show the patient’s status at the end of the study period. Outcomes were confirmed by examining individual-level claims data on ambulatory services.

### Data analysis

Continuous data are summarized as median (interquartile range [IQR]). Categorical data are expressed as frequency (percentage). For patients who received ECMO twice, only data from their first ECMO treatment were included in the analyses. Demographics, clinical characteristics, and year of treatment were compared by the Wilcoxon rank sum test or the chi-square test between patients who were discharged after ECMO and those who experienced in-hospital mortality. Reasons for ECMO were compared among age groups by the chi-square test. The LOS and total inpatient expenditure were compared by demographics and clinical characteristics using the Wilcoxon rank sum or by the Kruskal-Wallis test, with Bonferroni post-hoc pair-wise comparisons for type I error adjustment. To investigate factors associated with in-hospital mortality, crude and adjusted odds ratios (ORs) with 95% confidence intervals (CIs) were calculated using univariate and multivariate logistic regression.

For patients who were discharged from hospital after ECMO, survival analysis was carried out on the length of time between the date of discharge and date of death. Patients were censored from survival analysis if they were alive on 31 December 2010. Those who received ECMO twice during study period were excluded from the survival analysis. Kaplan-Meier curves were constructed with log-rank tests to detect differences in survival-after-discharge between groups by demographic and clinical characteristics and year of treatment. Cox proportional hazards regression models were constructed to determine crude and adjusted hazard ratios to investigate factors associated with mortality after discharge. Statistical analyses were performed using SAS software version 9.2 (SAS Institute Inc., Cary, NC, USA). Statistical significance was indicated by a two-tailed *P* < 0.05.

## RESULTS

### Demographic and clinical characteristics

The records of 3969 patients were included in the data analyses. Of these, 13 (0.33%) received ECMO twice and 3956 (99.67%) received ECMO once. The demographic and clinical characteristics of these patients are shown in Table 2. Most patients were aged between 40–64 years, were male, received ECMO at medical centers, and were treated for cardiovascular indications. The number of patients receiving ECMO increased over the study period, with increases being particularly profound during the last four years.

**Table 2.** Demographic and clinical characteristics of patients who received extracorporeal membrane oxygenation in Taiwan during 2000 to 2010 (*n* = 3969)

Characteristic	Total (*n* = 3969)	Hospital survivor (*n* = 1326)	Non-survivor (*n* = 2643)	*P* value
Age (year), median (IQR)	54.6 (35.7, 68.5)	47.7 (28.4, 61.1)	57.8 (41.3, 70.9)	<0.001^a^
Age group (year), *n* (%)				
<1	140 (3.5)	50 (35.7)	90 (64.3)	<0.001^b^
1–9	194 (4.9)	88 (45.4)	106 (54.6)	
10–17	142 (3.6)	63 (44.4)	79 (55.6)	
18–39	676 (17.0)	314 (46.4)	362 (53.6)	
40–54	867 (21.8)	325 (37.5)	542 (62.5)	
55–64	706 (17.8)	214 (30.3)	492 (69.7)	
65–74	716 (18.0)	189 (26.4)	527 (73.6)	
≥75	528 (13.3)	83 (15.7)	445 (84.3)	
Sex, *n* (%)				
Female	1315 (33.1)	470 (35.7)	845 (64.3)	0.028^b^
Male	2654 (66.9)	856 (32.3)	1798 (62.7)	
Calendar year, *n* (%)				
2000	52 (1.3)	15 (28.4)	37 (71.2)	<0.001^b^
2001	61 (1.5)	20 (32.8)	41 (67.2)	
2002	75 (1.9)	30 (40.0)	45 (60.0)	
2003	148 (3.7)	44 (29.7)	104 (70.3)	
2004	189 (4.8)	47 (24.9)	142 (75.1)	
2005	262 (6.6)	75 (28.6)	187 (71.4)	
2006	275 (6.9)	79 (28.7)	196 (71.3)	
2007	469 (11.8)	143 (30.5)	326 (69.5)	
2008	732 (18.4)	243 (33.2)	489 (66.8)	
2009	661 (16.7)	210 (31.8)	451 (68.2)	
2010	1045 (26.3)	420 (40.2)	625 (59.8)	
Calendar year^c^, *n* (%)				
2000–2002	188 (4.7)	65 (34.6)	123 (65.4)	<0.001^b^
2003–2008	2075 (52.3)	631 (30.4)	1444 (69.6)	
2009–2010	1706 (43.0)	630 (36.9)	1076 (63.1)	
Hospital setting, *n* (%)				
Medical Center	2862 (72.1)	967 (33.8)	1895 (66.2)	0.716^b^
Regional Hospital	1086 (27.4)	352 (32.4)	734 (67.6)	
District Hospital	21 (0.5)	7 (33.3)	14 (66.7)	
Major indications^d^, *n* (%)				
Cardiovascular	2729 (68.7)	909 (33.3)	1820 (66.7)	0.113^b^
Respiratory	710 (17.9)	245 (34.5)	465 (65.5)	
Trauma (injury)	150 (3.8)	60 (40)	90 (60)	
Other	380 (9.6)	112 (29.5)	268 (70.5)	

IQR, interquartile range.

^a^Determined by Wilcoxon rank sum test.

^b^Determined by chi-square test.

^c^Calendar years are divided into groups on the basis of National Health Insurance (NHI) policy: 2000–2002, NHI did not reimburse patients for extracorporeal membrane oxygenation (ECMO); 2003–2008, NHI reimbursed patients for specific/limited ECMO indications; 2009–2010, NHI reimbursed patients for extended ECMO indications.

^d^Major indication for ECMO was classified by ICD-9-CM code as follows: Cardiovascular: 394, 396, 397, 398, 410, 411, 414, 415, 416, 420, 421, 422, 423, 424, 425, 426, 427, 428, 429, 430, 435, 441, 442, 459, 745, 746, 747, 785; Respiratory: 12, 480, 481, 482, 483, 484, 485, 486, 487, 506, 507, 510, 514, 515, 518, 519, 748, 769, 770, 786, 986, 987; Trauma (injury): 800, 801, 807, 808, 852, 853, 854, 860, 861, 862, 868, 901. Others: 38, 270, 271, 273, 431, 432, 759, 768, 772, 775, 779, 962, 969, 972, 977, 980, 989, 994, 996, V420, V421, V422, V427, V431, V433, V59, unknown.

Table 2 also shows demographic and clinical characteristics for patients who were discharged after ECMO and for those who experienced in-hospital mortality. There were significant differences between patients who were discharged and those who died in hospital in the distribution of age (*P* < 0.001), sex (*P* = 0.028), and the proportion of patients treated by calendar year (*P* < 0.001). Of the 3969 patients who received ECMO, 1326 were discharged from hospital and 2643 died before discharge.

### Distribution of indications for ECMO by age group

**Figure 1.**
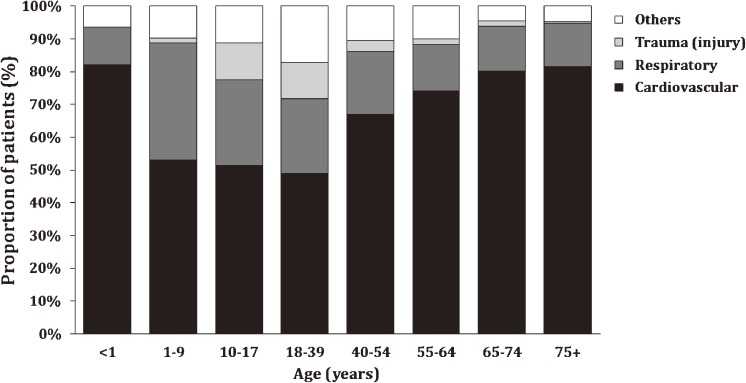
Summary of indications for extracorporeal membrane oxygenation (ECMO) by age categories group (<1, 1–9, 10–17, 18–39, 40–54, 55–64, 65–74, and ≥75 years) among patients who received ECMO in Taiwan during 2000 to 2010 (*n* = 3969).

The distributions of indications for ECMO differed significantly between the different age groups (*P* < 0.001, Figure 1). ECMO for cardiovascular disease was lowest for ages 18–39 years (49.0%) and highest for newborns (82.1%). Rates of ECMO for indications other than cardiovascular and respiratory disease and trauma (injury) were lowest among those aged 65–74 years (4.6%) and highest among those aged 18–39 years (17.2%).

### Length of hospital stay

**Figure 2.**
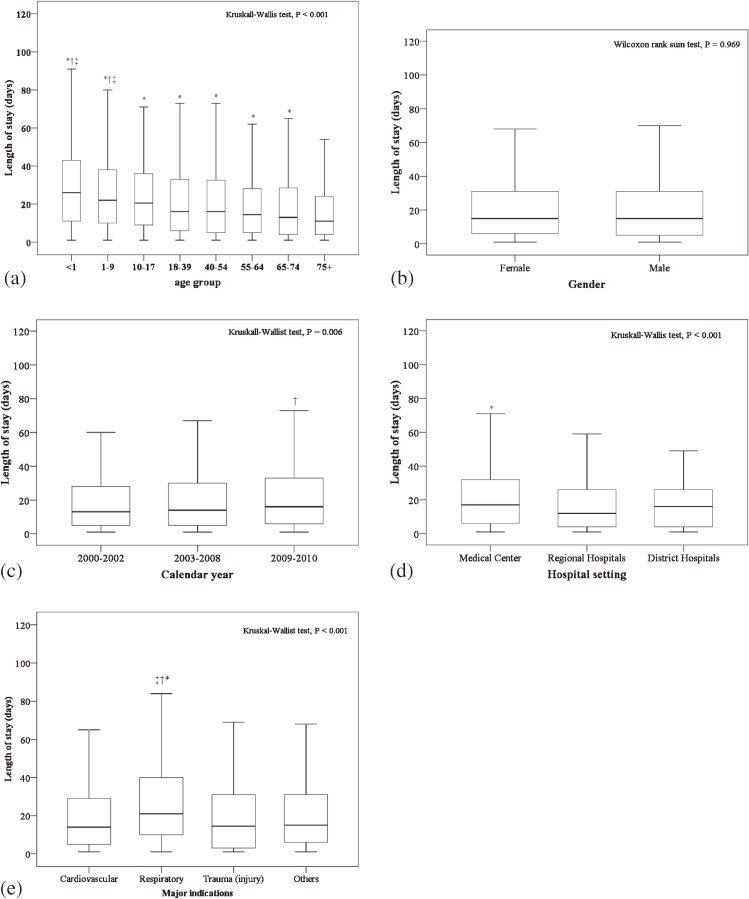
Length of hospital stay for all patients who received extracorporeal membrane oxygenation (ECMO) in Taiwan during 2000 to 2010 (*n* = 3969) by (a) age group, (b) sex, (c) calendar year, (d) hospital setting, and (e) indication for ECMO. Indicators of significant difference (*P* < 0.05) are defined as follows: (a) age group: *compared with ≥75 years, †compared with 65–74 years, ‡compared with 18–39 years; (b) sex: *compared with female; (c) calendar year: *compared with 2000–2002, †compared with 2003–2008; (d) hospital setting: *compared with regional hospitals, †compared with district hospitals; (e) indication for ECMO: *compared with cardiovascular disease, †compared with trauma (injury), ‡compared with other disease.

Among the 3969 patients who received ECMO (data from first ECMO treatment were included), the median LOS was 15 days (IQR, 5–31 days). The LOS results are summarized by demographic and clinical characteristics in Figure 2. The median LOS was significantly different among the different age groups (*P* < 0.001), calendar years (*P* = 0.006), hospital settings (*P* < 0.001), and the indications for ECMO (*P* < 0.001). Elderly patients had significantly shorter median LOS than the other age groups (Figure 2a). Patients who received ECMO during 2009–2010 had a significantly longer median LOS than those who received ECMO during 2003–2008 (Figure 2c). Patients treated in medical centers had a significantly longer median LOS than those treated in regional hospitals (Figure 2d). Patients who required ECMO for respiratory disease had a longer median LOS than those who required ECMO for all other indications (Figure 2e).

### Total inpatient expenditure

**Figure 3.**
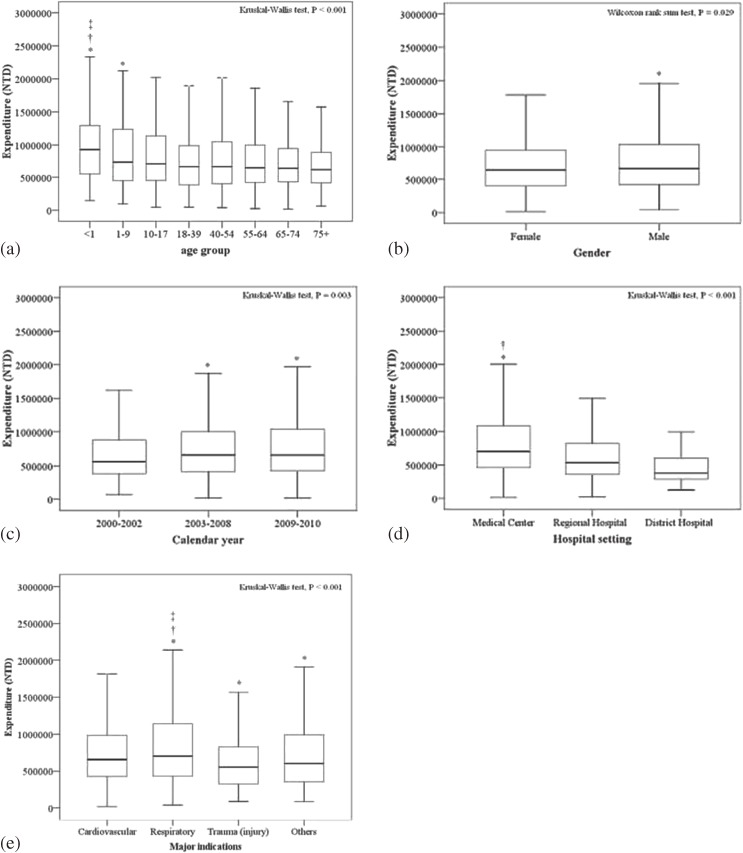
Total inpatient expenditure of patients who received extracorporeal membrane oxygenation (ECMO) in Taiwan during 2000 to 2010 (*n* = 3969) by (a) age group, (b) sex, (c) calendar year, (d) hospital level, and (e) indication for ECMO. Indicators of significant difference (*P* < 0.05) are defined as follows: (a) age group: *compared with ≥75 years, †compared with 65–74 years, ‡compared with 18–39 years; (b) sex: *compared with female; (c) calendar year: *compared with 2000–2002, †compared with 2003–2008; (d) hospital setting: *compared with regional hospitals, †compared with district hospitals; (e) indication for ECMO: *compared with cardiovascular disease, †compared with trauma (injury), ‡compared with other disease.

Among 3969 patients who received ECMO (data from first ECMO treatment were included), the median total inpatient expenditure was 655 607 New Taiwan Dollars (NTD) (IQR, 414 437–1 004 288 NTD). The average exchange rate for the 1996 to 2010 period was 1 United States (U.S.) Dollar (USD) to 31.64 NTD. The total inpatient expenditure results are summarized by demographic and clinical characteristics in Figure 3. Median total inpatient expenditure was significantly different among the different age groups (*P* < 0.001), sexes (*P* = 0.029), calendar years (*P* = 0.003), hospital settings (*P* < 0.001), and indications for ECMO (*P* < 0.001). Median expenditure was significantly higher for newborns than patients aged 18 to 39 years, 65 to 74 years, and ≥75 years (Figure 3a). Male patients had significantly higher median expenditure than female patients (Figure 3b). Patients who received ECMO during 2003–2008 and 2009–2010 had significantly higher median expenditure than those who received ECMO during 2000–2002 (Figure 3c). Patients treated at medical centers had significantly higher median expenditure than those treated at regional and district hospitals (Figure 3d). Patients who received ECMO for trauma had significantly lower median expenditure than those who received ECMO for all other indications (Figure 3e). [In order for the international readers to understand the scale of expenditure on ECMO use in Taiwan, a reference of medical cost in Taiwan is as follows: ≥2 vessels coronary artery bypass without diagnostic coronary angiography: 14 000 USD; intensive care unit cost/day: 213 USD (ward and nursing fee).]

### Survival-to-discharge and factors associated with in-hospital mortality

The survival-to-discharge rates are summarized by different demographic and clinical characteristics in Table 3. The overall survival-to-discharge rate was 33.4%. The survival-to-discharge rate was lowest for the elderly, slightly higher in females than males, highest for patients treated during 2009–2010, highest for patients treated in medical centers, and highest for patients who required treatment for trauma.

**Table 3.** Univariate and multivariate logistic regression models for factors associated with in-hospital mortality among patients who received extracorporeal membrane oxygenation in Taiwan during 2000 to 2010 (*n* = 3969)

Characteristic	*n*	Survival-to-discharge^a^ (%)	Univariate analysis		Multivariate analysis	
	
			Crude OR	*P* value	Adjusted OR	*P* value
Age group (years)						
<1	140	35.7	1.44 (0.89, 2.32)	0.139	1.52 (0.94, 2.47)	0.090
1–9	194	45.4	0.96 (0.62, 1.49)	0.856	0.96 (0.62, 1.49)	0.858
10–17	142	44.4	1.00 (reference)	—	1.00 (reference)	—
18–39	676	46.4	0.92 (0.64, 1.32)	0.651	0.92 (0.64, 1.33)	0.646
40–54	867	37.5	1.33 (0.93, 1.90)	0.119	1.40 (0.97, 2.02)	0.070
55–64	706	30.3	1.83 (1.27, 2.65)	0.001	1.98 (1.36, 2.89)	<0.001
65–74	716	26.4	2.22 (1.54, 3.22)	<0.001	2.46 (1.68, 3.58)	<0.001
≥75	528	15.7	4.28 (2.85, 6.41)	<0.001	4.71 (3.11, 7.12)	<0.001
Sex						
Female	1315	35.7	1.00 (reference)	—	1.00 (reference)	—
Male	2654	32.3	1.17 (1.02, 1.34)	0.028	1.11 (0.96, 1.29)	0.146
Calendar year						
2000–2002	188	34.6	1.11 (0.81, 1.52)	0.525	1.32 (0.95, 1.84)	0.094
2003–2008	2075	30.4	1.34 (1.17, 1.54)	<0.001	1.43 (1.24, 1.64)	<0.001
2009–2010	1706	36.9	1.00 (reference)	—	1.00 (reference)	—
Hospital setting						
Medical Center	2862	33.8	1.00 (reference)	—	1.00 (reference)	—
Regional Hospital	1086	32.4	1.06 (0.92, 1.24)	0.413	0.98 (0.84, 1.15)	0.825
District Hospital	21	33.3	1.02 (0.41, 2.54)	0.965	0.84 (0.32, 2.17)	0.716
Major indications						
Cardiovascular	2729	33.3	0.84 (0.66, 1.06)	0.136	0.62 (0.48, 0.79)	<0.001
Respiratory	710	34.5	0.79 (0.61, 1.04)	0.092	0.76 (0.57, 1.00)	0.047
Trauma (injury)	150	40	0.63 (0.42, 0.93)	0.020	0.72 (0.48, 1.07)	0.106
Other	380	29.5	1.00 (reference)	—	1.00 (reference)	—

OR, odds ratio; CI, confidence interval.

^a^Survival-to-discharge = number of hospital survivors/number of total population.

The factors associated with in-hospital mortality are also summarized in Table 3. After adjusting for age, sex, calendar year, hospital setting, and major indication for ECMO, multivariate logistic regression revealed that older age (≥75 years vs 10–17 years; 65–74 years vs 10–17 years; 55–64 vs 10–17 years) and earlier calendar year (2003–2008 vs 2009–2010) were associated with a significantly higher risk of in-hospital mortality (all *P* < 0.001). In contrast, the requirement of ECMO for cardiovascular and respiratory was associated with a significantly lower risk of in-hospital mortality than ECMO for other diseases (*P* < 0.001).

### Survival-after-discharge and factors associated with mortality after discharge

**Figure 4.**
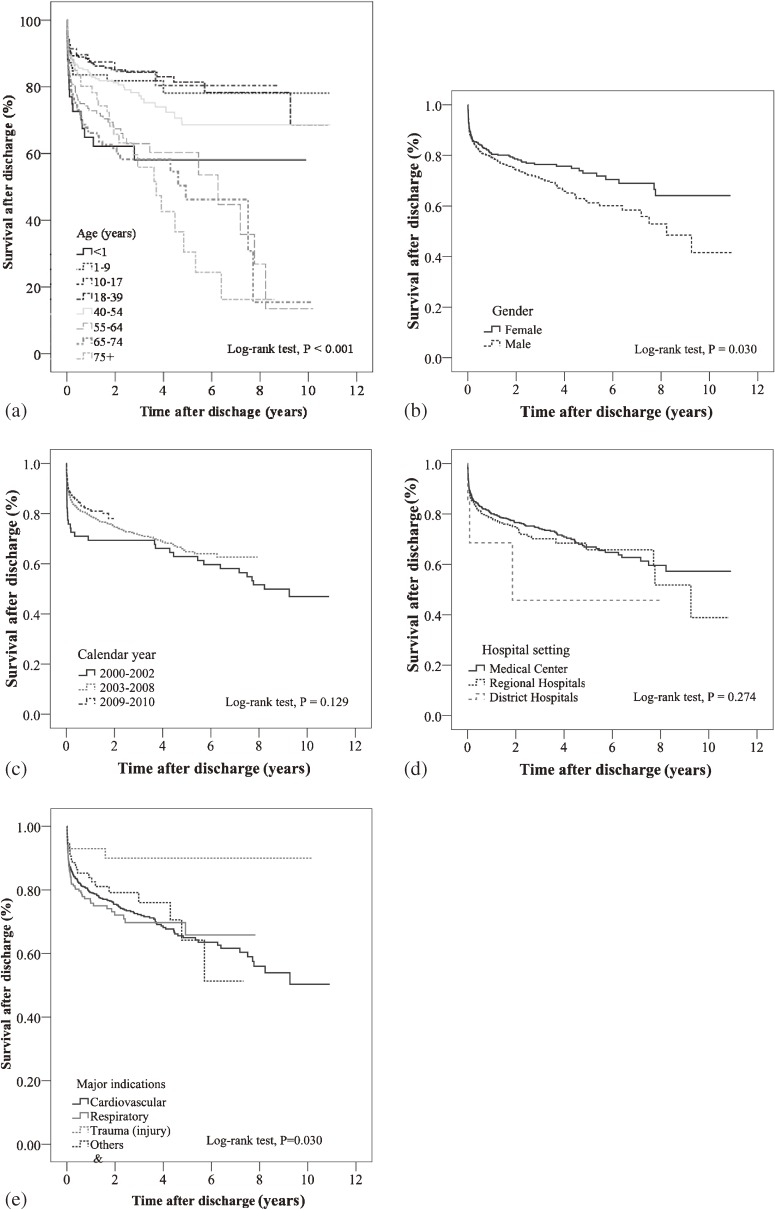
Kaplan-Meier curves of survival-after-discharge in patients who received extracorporeal membrane oxygenation (ECMO) in Taiwan during 2000 to 2010 (*n* = 1313) by (a) age group, (b) sex, (c) calendar year, (d) hospital level, and (e) indication for ECMO.

A total of 1313 patients who were received ECMO and were discharged were included in the survival-after-discharge analyses. The median length of follow-up after discharge was 15.0 months (IQR, 2.7–34.4 months). The 1-year and 5-year survival-after-discharge rates were 79.6% and 66.2%, respectively. The 1-year and 5-year survival-after-discharge rates are summarized by different demographic and clinical characteristics in Table 4. The 1-year survival-after-discharge rate was lowest for newborns, higher for females than males, highest for patients treated during 2009–2010, highest for patients treated in medical centers, and highest for patients who required treatment for trauma. Figure 4 shows the corresponding K-M curves for identifying the association of survival-after-discharge with those characteristics. There were significant differences in survival-after-discharge curves by age (*P* < 0.001), sex (*P* = 0.030), and indications for ECMO (*P* = 0.030) (Figure 4).

**Table 4.** Univariate and multivariate Cox’s proportional hazard models for factors associated with mortality after discharge among patients who received extracorporeal membrane oxygenation in Taiwan during 2000 to 2010 (*n* = 1313)

Characteristic	*n*	Survival-after-discharge^a^		Univariate analysis		Multivariate analysis	
		
		1-year (%)	5-year (%)	Crude HR	*P* value	Adjusted HR	*P* value
Age group (years)							
<1	49	64.9	58.0	2.81 (1.26, 6.26)	0.011	2.83 (1.27, 6.33)	0.011
1–9	87	83.6	78.1	1.25 (0.55, 2.83)	0.590	1.09 (0.48, 2.47)	0.841
10–17	60	87.5	80.4	1.00 (reference)	—	1.00 (reference)	—
18–39	313	87.2	81.4	1.07 (0.52, 2.18)	0.863	1.06 (0.52, 2.17)	0.876
40–54	322	83.4	68.6	1.53 (0.76, 3.08)	0.232	1.46 (0.72, 2.94)	0.295
55–64	211	72.9	60.3	2.80 (1.40, 5.63)	0.004	2.78 (1.38, 5.63)	0.004
65–74	188	66.2	46.2	3.25 (1.62, 6.52)	0.001	3.11 (1.54, 6.27)	0.002
≥75	83	80.1	30.4	3.15 (1.50, 6.63)	0.002	3.04 (1.44, 6.42)	0.004
Sex							
Female	466	80.4	72.9	1.00 (reference)	—	1.00 (reference)	—
Male	847	79.1	61.2	1.30 (1.03, 1.64)	0.030	1.24 (0.97, 1.57)	0.086
Calendar year							
2000–2002	62	69.4	62.9	1.53 (0.98, 2.38)	0.060	1.80 (1.15, 2.84)	0.011
2003–2008	622	78.9	64.8	1.21 (0.94, 1.57)	0.142	1.31 (1.01, 1.70)	0.041
2009–2010	629	81.4	NA	1.00 (reference)	—	1.00 (reference)	—
Hospital setting							
Medical Center	956	80.0	66.9	1.00 (reference)	—	1.00 (reference)	—
Regional Hospital	350	78.6	65.8	1.14 (0.89, 1.46)	0.302	1.05 (0.82, 1.35)	0.689
District Hospital	7	68.6	45.7	2.09 (0.67, 6.53)	0.206	2.07 (0.65, 6.58)	0.218
Major indications							
Cardiovascular	898	79.2	65.0	1.19 (0.78, 1.83)	0.427	0.90 (0.58, 1.39)	0.627
Respiratory	245	75.8	65.8	1.29 (0.80, 2.09)	0.296	1.32 (0.81, 2.15)	0.258
Trauma (injury)	60	93.0	90.0	0.36 (0.14, 0.96)	0.040	0.36 (0.13, 0.95)	0.039
Other	110	83.9	64.2	1.00 (reference)	—	1.00 (reference)	—

HR, hazard ratio; CI, confidence interval.

^a^Survival-after-discharge was estimated using the Kaplan-Meier approach.

The factors associated with mortality after discharge are summarized in Table 4. After adjusting for age, sex, calendar year, hospital setting, and major indication for ECMO, multivariate Cox proportional hazards model analysis revealed that newborn age (vs 10–17 years), 55–64 years (vs 10–17 years), 65–74 year (vs 10–17 years), and elderly age (vs 10–17 years), as well as earlier calendar year (2000–2002 vs 2009–2010; 2003–2008 vs 2009–2010) were associated with a significantly higher risk of death after discharge (all *P* < 0.05). In contrast, the requirement of ECMO for trauma was associated with a significantly lower risk of death after discharge compared to other diseases (*P* = 0.039).

## DISCUSSION

In this retrospective study, we found that the use of ECMO increased markedly in Taiwan between 2000 and 2010, particularly after 2002 (December), when the NHI began reimbursing the costs of ECMO. The overall mortality rate for patients who received ECMO was high (>70%) but did decrease between 2000 and 2010. This decrease in mortality may reflect technological advances and/or misuse of ECMO for treating patients not sufficiently critical to warrant the treatment. We also found that a number of factors were associated with survival outcomes in patients who received ECMO, most notably age and indication for ECMO. These findings represent the first systematic examination of ECMO use in Taiwan.

Patients who required ECMO for respiratory disease had longer LOS than other patients. LOS was calculated from the first day of hospital admission in all patients, not from the day on which ECMO was begun, so it is possible that respiratory disease patients had more severe disease from the beginning, or that they needed a longer stay because they needed a period of assisted ventilation before and after ECMO use. Elderly patients had a significantly shorter LOS than other age groups. Non-survival patients were included in the LOS analysis, which may explain the shorter LOS of elderly patients.

We found that the increase in ECMO use was extraordinarily profound between 2006 and 2010. There are several explanations for this increase. First, there were several very high profile cases in 2006 involving successful ECMO treatment that were widely reported in the media. Until that time, very few people in Taiwan were aware of ECMO. Indeed, ECMO became so widely publicized in Taiwan that patients and their families asked for “Dr. ECMO’s” help for the treatment of critical conditions, regardless of the clinical indication.^14^ Second, there was an increase in ECMO supply by hospitals and doctors during this time period. Previously, ECMO devices and operating teams were only available at large-scale medical centers (there are 19 of these medical centers and 74 regional hospitals in Taiwan). However, with the increased media exposure and growing demand from patients, many regional hospitals began offering ECMO. A few large-scale district hospitals also began offering ECMO. Around this time, Chen et al published findings suggesting that extracorporeal cardiopulmonary resuscitation (CPR) provided a survival benefit over conventional CPR in patients with in-hospital cardiac arrest of cardiac origin.^7^ This publication undoubtedly increased doctors’ and hospitals’ willingness to use ECMO. A third reason behind the increase in ECMO use between 2000 and 2010 is changes in NHI reimbursement criteria. Typically, NHI beneficiaries are required to provide 10% copayments for all inpatient expenses. However, such copayments can be waived if patients are diagnosed with a major illness (under defined category) or are admitted to an intensive care unit due to a defined critical condition. Removing the financial burden presumably prompts patients’ families to push for expensive treatments, such as ECMO, that they would otherwise not be able to afford. The large jump in ECMO use between 2009 and 2010 is a reflection of the extended NHI indications for ECMO that were implemented in 2009.

Overall, the results from our study indicate that fewer than 30% of patients who received ECMO in Taiwan between 2000 and 2010 survived. We believe that this indicates that ECMO may be being used in Taiwan in clinical situations too severe for ECMO to produce any added benefit, particularly among older adults, for whom the survival rate is lower than that of the pediatric population. The survival rate for patients aged 65 or higher was approximately 20%. Worryingly, use among adults significantly increased between 2000 and 2010, whereas use among the pediatric population decreased as a proportion of the study population. Only 13.1% of our study population was aged <18 years. This contrasts quite dramatically with data from the Extracorporeal Life Support Registry Report, in which 92.9% (35 030) of patients from over 170 active centers within and outside of the U.S. were aged <18 years.^15^ Although there is now support from a randomized controlled trial for the use of ECMO in treating severe acute respiratory failure in adults,^5^ there are no such data from well-designed studies to support the use of ECMO for treating cardiac failure in adults. Hence, we suggest that the indications for use of ECMO in adults in Taiwan are too broad, which has led to use in situations where it does not produce substantial added benefit.

In a previous study, Bartlett et al examined ECMO use between 1980 and 1998 at a single U.S. institution and found that survival-to-discharge in moribund patients with respiratory failure was 88% in 586 neonates, 70% in 132 children, and 56% in 146 adults.^16^ Survival in moribund patients with cardiac failure was 48% in 105 children and 33% in 31 adults. Although the survival rates in our study are lower than the rates reported by Bartlett et al, our age-related survival trends are consistent in that survival was better in younger patients than older ones. We suggest that the lower rates of survival in Taiwan may be at least in part due to clinically unjustifiable ECMO use; that is, some patients who received treatment were not going survive, regardless of any intervention. Being able to identify such patients before ECMO use would be of obvious benefit, sparing the healthcare system the undue cost and the patient undue suffering and potential complications. To this end, our regression analyses revealed that patient age and indication for treatment were significantly associated with measures of mortality. As such, these variables should be considered when evaluating candidate patients for ECMO.

Unwarranted use of ECMO has a number of intangible costs that should be considered. The procedure itself is not without risk; hence, patients may suffer complications without any likelihood of experiencing significant benefit. Patients’ quality of life should also be considered. In some cases, ECMO may serve to extend life without any real hope of recovery. In such instances, patients who may have been suitable organ donors may be less suitable or unsuitable after ECMO usage.

Our study has a number of limitations. First, we were unable to perform any cost-effectiveness analyses because the NHI database does not provide the exact date of ECMO. Second, we did not perform corrections for inflation when evaluating expenditures. Third, we were unable to examine severity of disease, amount of time on ECMO, complications after ECMO, or comorbidities after discharge because this information is not available in NHI claims data. Finally, we do not have data from matched groups of non-ECMO patients, so we cannot compare survival, LOS, cost, and other parameters between ECMO and non-ECMO treatments.

In summary, the findings from our study reveal that use of ECMO increased markedly in Taiwan between 2000 and 2010. The reasons for this increase are complicated but likely include extension of reimbursement criteria, increased availability, and increased patient demand. We believe the data presented herein indicate that ECMO is currently being used more than medically or economically justifiable in Taiwan, particularly among the adult population, who experience a less pronounced survival benefit than pediatric patients. Such overuse increases costs and draws resources from other interventions/treatments that may be more beneficial and/or cost-effective. A key reason for the apparent overuse of ECMO in Taiwan is that the indications for ECMO are broad, much more so than elsewhere.^15^^,^^16^ We suggest that the clinical indications for ECMO in Taiwan be re-evaluated with reference to established guidelines followed in other countries. Further, our findings suggest that patient age and clinical indication for treatment are particularly important factors that should be considered when evaluating whether a patient should receive ECMO.
